# Increased incidence of age-related macular degeneration in sensorineural hearing loss: A population-based cohort study

**DOI:** 10.1371/journal.pone.0222919

**Published:** 2019-10-23

**Authors:** Chia-Yi Lee, Hung-Chi Chen, Pei-Hsuan Wu, Jessie Chao-Yun Chi, Chi-Chin Sun, Jing-Yang Huang, Hung-Yu Lin, Shun-Fa Yang

**Affiliations:** 1 Department of Ophthalmology, Show Chwan Memorial Hospital, Changhua, Taiwan; 2 Department of Optometry, College of Medicine and Life Science, Chung Hwa University of Medical Technology, Tainan, Taiwan; 3 Department of Ophthalmology, Chang Gung Memorial Hospital, Linkou, Taiwan; 4 Department of Medicine, Chang Gung University College of Medicine, Taoyuan, Taiwan; 5 Center for Tissue Engineering, Chang Gung Memorial Hospital, Linkou, Taiwan; 6 Department of Otolaryngology–Head and Neck Surgery, Tri-Service General Hospital, Taipei, Taiwan; 7 Institute of Medicine, Chung Shan Medical University, Taichung, Taiwan; 8 Department of Otorhinolaryngology Head and Neck Surgery, Taichung Hospital, Ministry of Health and Welfare, Taichung, Taiwan; 9 Department of Ophthalmology, Chang Gung Memorial Hospital, Keelung, Taiwan; 10 Department of Chinese Medicine, Chang Gung University, Taoyuan City, Taiwan; 11 Department of Medical Research, Chung Shan Medical University Hospital, Taichung, Taiwan; 12 Department of Optometry, Chung Shan Medical University, Taichung, Taiwan; 13 Department of Exercise and Health Promotion, Chung Chou University of Science and Technology, Changhua, Taiwan; University of Florida, UNITED STATES

## Abstract

**Background:**

To evaluate the incidence of age-related macular degeneration (AMD) in patients diagnosed with sensorineural hearing loss (SNHL) via the application of the National Health Insurance Research Database in Taiwan.

**Methodology/Principal findings:**

A retrospective cohort study was conducted. Patients with a diagnosis of SNHL was enrolled in the study group after exclusion and a propensity score matched group without SNHL was served as the control group with a 1:2 ratio. The main outcome was regarded as the emergence of AMD diagnostic codes. Cox proportional hazard regression was applied to analyze the incidence and adjusted hazard ratio (aHR) of AMD in the multivariate model. A total of 15,686 patients with SNHL were included in the study group while another 31,372 non-SNHL patients served as the control group. After a follow-up interval up to 16 years, there were 484 AMD events occurred in the study group and 660 AMD cases in those non-SNHL patients with a significantly higher aHR compared to the control group after adjusting for multiple potential risk factors (aHR: 1.399, 95% CI: 1.244–1.574). Other prominent risk factors for AMD included older age, ischemic heart disease, hyperlipidemia, Alzheimer's disease, liver disease and kidney disease. Besides, a higher cumulative probability of AMD was observed in the study group (log-rank *P* <0.0001).

**Conclusion:**

The patients with SNHL demonstrated a higher incidence of developing AMD.

## Introduction

Sensorineural hearing loss (SNHL) is the hearing impairment causing by damage of cochlear, labyrinth and central nervous system.[1, 2] Concerning the epidemiology, the SNHL affect the majority of population for which the prevalence of age-related SNHL up to 60 percent in the elderly aged more than 65 years.[3] About the etiology of SNHL, various etiologies including noise-related, infectious, autoimmune ear disease, drug toxicity and vascular lesions can lead to SNHL.[2, 4] In addition, central nervous system disease such as multiple sclerosis can also induce the development of SNHL.[5]

Several neurodegenerative disorders are associated with SNHL concurrently according to previous experiences.[6] Alzheimer’s disease, a dementia characterized with memory impairment and executive dysfunction which presented with of amyloid and tau,[7] revealed a higher risk of SNHL occurrence.[8] In addition, the SNHL has been illustrated to be associated with other brain disorders including cognitive impairment and general dementia in different studies.[9] Since the above neurological diseases share the similar clinical manifestation of neurodegenerative process with SNHL,[2, 7, 9] a general neurodegenerative impairment involving other organ in SNHL population may exist.

Age-related macular degeneration (AMD) is a chronic and degenerative ocular disorder that characterizes with progressively atrophies of choroid, retina pigment epithelium and photoreceptors, mainly involve the central macular region.[10, 11] In a previous study, higher occurrence of age-related SNHL with poorer pure-tone average was observed in those with AMD.[12] However, the whole study population in the previous study is only 93 participants and only the visual and auditory data were analyzed. As a result, a population-based research with large study number and multiple potential risk factors should be conducted.

The aim of current study is to evaluate the incidence of AMD in patients with SNHL via the use of the National Health Insurance Research Database (NHIRD) in Taiwan. In addition, potential risk factors including several neurodegenerative diseases were also analyzed in the multivariable model.

## Materials and method

### Data source

This retrospective population-based cohort study was approved by the National Health Insurance Administration and the Institutional Review Board (IRB) of Chung Shan Medical University and the IRB waived the requirement for informed consent. Provided by the Taiwan National Health Research Institutes, the NHIRD contains data of insurance claims from more than 99% of Taiwan’s population. The claims data were obtained from the Longitudinal Health Insurance Database 2005 version (LHID 2005) in the current study. The LHID 2005 contains data on two million patients randomly sampled from the NHIRD registry for the year 2005. The LHID 2005 data were linked from 1 January 2000, to 31 December 2016, and both the International Classification of Diseases, Ninth Revision (ICD-9) and International Classification of Diseases, Tenth Revision (ICD-10) were used for disease diagnosis. Details on the medications prescribed for the patients and the demographics, socioeconomic status, and residence of the patients are also available in the NHIRD.

### Patient selection

Patients were defined as having SNHL if their medical records indicated (1) a diagnosis of SNHL (ICD-9 codes: 389.1x, 389.2x, ICD-10 codes: H90.3, H90.4x, H90.5, H90.6, H90.7x, H90.8, H90.A2x, H90.A3x, H91.2x), (2) the arrangement of pure-tone audiogram (procedure codes: 22001C) before the diagnosis of SNHL, and (3) receipt of the SNHL diagnosis by an otorhinolaryngologist (department code: 09). The index date was set as the date of the diagnosis of SNHL. To more accurately elucidate the association between SNHL and AMD, the following exclusion criteria were applied to exclude certain impaired ocular and otological conditions: (1) receipt of a diagnosis of legal blindness (ICD-9 codes: 369.4, ICD-10 codes: H54.0x, H54.1x, H54.4x, H54.8) at any time; (2) receipt a diagnosis of ocular tumors (ICD-9 codes: 190.0–190.9, ICD-10 codes: C69.x) before the index date; (3) receipt of any type of eyeball removal surgery or diagnosed as anophthalmos (ICD-9 codes: 16.3x, 16.4x, 16.5x, 871.3, ICD-10 codes: Q11.1, S05.7x, Z90.01 plus procedure codes: 85001C, 85002C, 86808B) before the index date; (4) receipt a diagnosis of deafness (ICD-9 codes: 389.7, ICD-10 codes: H91.3); (5) receipt a diagnosis of otological tumors (ICD-9 codes: 160.1, ICD-10 codes: C30.1) before the index date; (6) receipt of any type of labyrinth removal surgery before the index date; (7) receipt a diagnosis of any type of glaucoma and glaucoma suspect (ICD-9 codes: 365.x, ICD-10 codes: H40.x, H42.x), optic neuropathy (ICD-9 codes: 377.x, ICD-10 codes: H46.x, H47.x) and AMD (diagnostic codes are shown in the following section) before the index date; (8) the diagnosis of SNHL was earlier than 2005, and (9) age younger than 20 or older than 100. In addition, each individual in the study group was propensity score-matched with two non-SNHL individuals, as discussed in the following sections, which constituted the control group. Patients with SNHL who could not be matched with non-SNHL patients were excluded.

### Main outcome measurement

The development of AMD was regarded as the main outcome in the current study which was based on the emergence of AMD-related diagnostic codes (ICD-9 codes: 362.50, 362.51, 362.52, ICD-10 codes: H35.30, H35.31x, H35.32x) after the index date, and (2) the receipt of optical coherence tomography exam (procedure code: 23506C) before AMD diagnosis. Although diagnosis of “retina edema” and “retinal pigment epithelium detachment” may be associated with some AMD, these diagnostic codes were eliminated from the current study to prevent overestimation and confusion. Furthermore, only patients who received the abovementioned diagnostic codes by an ophthalmologist (department code: 10) were considered as having achieved an outcome and were included in the study.

### Demographic variables and co-morbidities

To make the health condition of participants more homogenous, we also considered the effects of age, gender and the following systemic co-morbidities, according to our Modified Deyo–Charlson co-morbidity index in the multivariate analysis model: hypertension (ICD-9 codes: 401.x-405.x, ICD-10 codes: I10, I11.x, I13.x, I15.x, I16.x, I87.3x, I97.3x, O10.x, O11.x, O13.x, O16.x), diabetes mellitus (ICD-9 codes: 250.x, 277.7, ICD-10 codes: O24.4, E11.x, E13.x, E88.81), ischemic heart diseases (ICD-9 codes: 410.x, 412.x, 414.0, 414.0x, 414.2, 414.3, 414.4, 414.8, 414.9, ICD-10 codes: I20.x-I25.x), hyperlipidemia (ICD-9 codes: 272.0, 272.1, 272.2, 272.4, 272.9, ICD-10 codes: E78.0x, E78.1, E78.2, E78.3, E78.4x, E78.5, E78.70, E78.79, E78.89, E78.9), congestive heart failure (ICD-9 codes: 398.91, 402.01, 402.11, 402.91, 404.01, 404.03, 404.11, 404.13, 404.91, 404.93, 425.4–425.9, 428.x, ICD-10 codes: I50.2x, I50.3x, I50.4x, I50.84, I50.89, I50.9), cerebrovascular disease (ICD-9 codes: 362.34, 430.x–438.x, ICD-10 codes: G46.x, I60.x-I66.x, I67.0, I67.1, I67.2, I67.6, I67.81, I67.82, I67.84x, I67.89, I67.9), dementia (ICD-9 codes 290.x, 294.1, 331.2, ICD-10 codes: F01.x, F02.x, F03.x, G31.x), Alzheimer's disease (ICD-9 codes: 331.0, ICD-10 codes: G30.x), Parkinson's disease (ICD-9 codes: 332.0, ICD-10 codes: G20, G21.x), liver disease (ICD-9 codes: 070.22, 070.23, 070.32, 070.33, 070.44, 070.54, 070.6, 070.9, 456.0–456.2, 570.x, 571.x, 572.2–572.8, 573.3, 573.4, 573.8, 573.9, V42.7, ICD-10 codes: K72.x-K77, T86.43, T86.49, Z94.4), rheumatic disease (ICD-9 codes: 446.5, 710.0, 710.1, 710.3, 710.4, 714.0–714.2, 714.8, 725.x, ICD-10 codes: M05.1x, M05.2x-M05.9, M31.6, M32.1x, M32.8, M32.9, M33.03, M33.13, M33.2x, M33.90, M33.93, M34.0x, M34.1x, M34.9, M35.3), kidney disease (ICD-9 codes: 403.x, 404.x, 582.x, 583.0–583.7, 585.x, 586.x, 588.x, V42.0, V45.1, V56.x, ICD-10 codes: E08.2x, E09.2x, E11.2x, E13.2x, I12.x, I13.1, N03.x, N04.x, N11.x, N18.x, O10.2x, O10.3x, Z49.31, I95.3, T82.43XA, E85.3, R88.0, T82.4x), and hemiplegia or paraplegia (ICD-9 codes: 334.1, 342.x, 343.x, 344.0–344.6, 344.9, ICD-10 codes: G80.0-G80.2, G80.8, G80.9, G81.x, I69.33x-I69.36x, I69.83x-I69.86x, I69.93x- I69.96x). We longitudinally traced the data from the index date until the date of AMD diagnosis, withdrawal from the National Health Insurance program, or 31 December 2016.

### Statistical analysis

SAS version 9.4 (SAS Institute Inc, NC, USA) was employed for all the analyses. After propensity-matching with 1:2 ratios of the study and control groups, the incidence rate and corresponding 95% confidence intervals (CI) were calculated using Poisson regression. Multiple Cox proportional hazard regression was adopted to compute adjusted hazard ratios (aHR) by incorporating the aforementioned demographic data, and systemic co-morbidities in the multivariate model. The aHR of all demographic data and systemic co-morbidities were also analyzed. In the next step, the subgroup analysis according to the age-, gender- and duration of SNHL-based subgroups in the study group was conducted. We plotted Kaplan–Meier curve to indicate the cumulative incidence proportion of AMD between the study and control groups, and used the log rank test to determine the significant difference between the survival curves. Because most patients in the NHIRD are Han Taiwanese, race was not considered as a covariate. Statistical significance was set at *P* < 0.05. A *p* value lesser than 0.0001 was depicted as *P* <0.0001.

## Results

**Fig 1:**
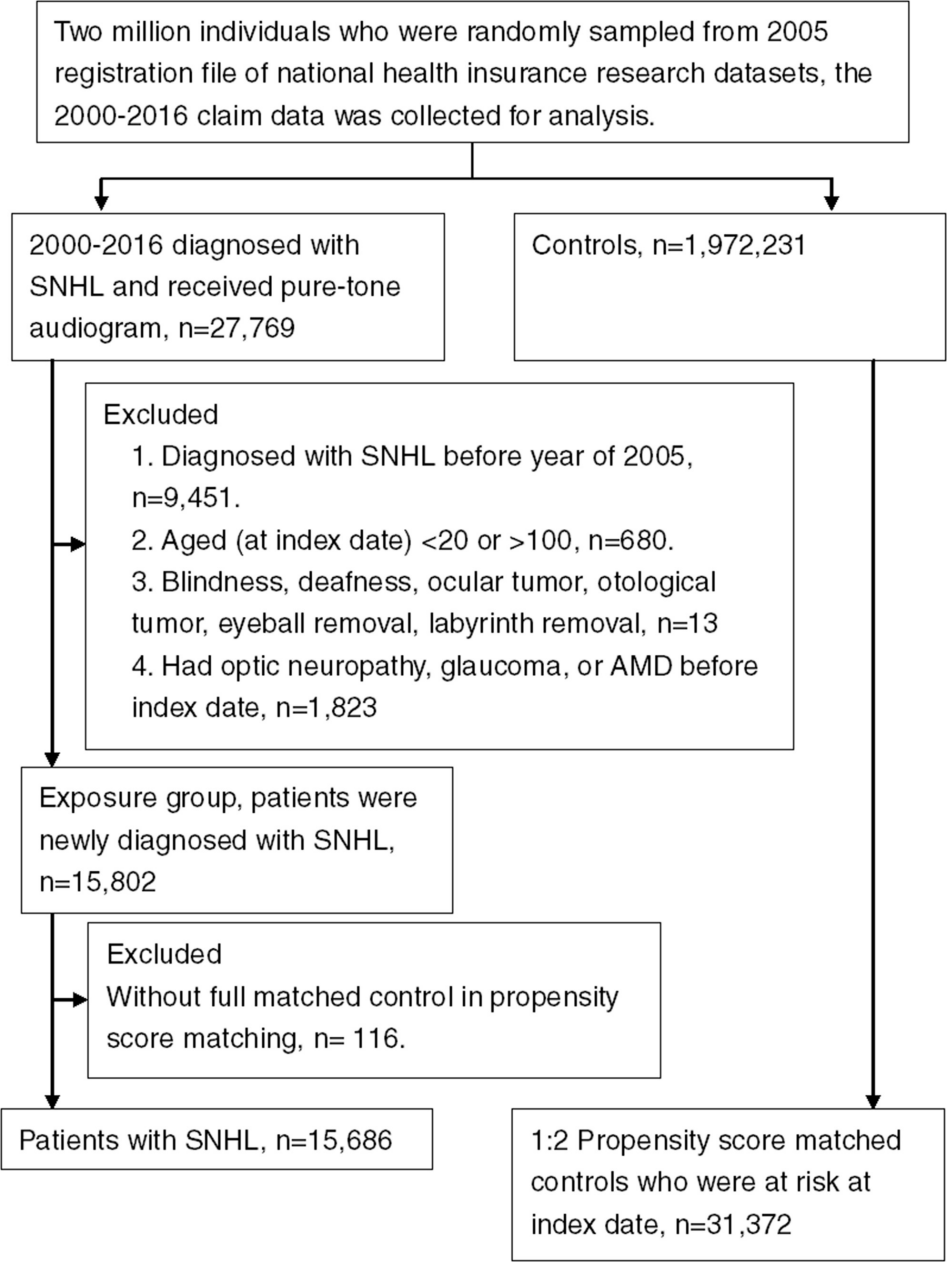
The flowchart of subject selection. SNHL: sensorineural hearing loss.

A total of 15,686 patients with SNHL were included in the study group while another 31,372 non-SNHL patients served as the control group. The flow chart of patient selection was shown in Fig 1. The numbers of Parkinson's disease was significantly higher in the study group and the numbers of hypertension was significantly higher in the control group, while the remaining basic characters involved age and gender distributions remained similar (Table 1).

**Table 1:** Basic characters between the study and control groups.

Basic characters	Study	Control	*P* value
Age			0.2296
<40	1987 (12.67%)	3839 (12.24%)	
40–59	5338 (34.03%)	10518 (33.53%)	
60–79	6542 (41.71%)	13362 (42.59%)	
> = 80	1819 (11.6%)	3653 (11.64%)	
Sex			0.7436
Male	8480 (54.06%)	17010 (54.22%)	
Female	7206 (45.94%)	14362 (45.78%)	
Co-morbidities (before index date)			
Hypertension	7755 (49.44%)	15826 (50.45%)	0.0394
Diabetes mellitus	3842 (24.49%)	7777 (24.79%)	0.4821
Ischemic heart diseases	3821 (24.36%)	7807 (24.89%)	0.2124
Hyperlipidemia	5849 (37.29%)	11719 (37.35%)	0.8875
Heart failure	1487 (9.48%)	3109 (9.91%)	0.1383
Cerebrovascular disease	3103 (19.78%)	6199 (19.76%)	0.9543
Dementia	536 (3.42%)	1003 (3.2%)	0.2060
Alzheimer's disease	49 (0.31%)	72 (0.23%)	0.0942
Parkinson's disease	231 (1.47%)	365 (1.16%)	0.0047
Liver disease	5218 (33.27%)	10513 (33.51%)	0.5947
Rheumatic disease	639 (4.07%)	1237 (3.94%)	0.4945
Kidney disease	6994 (44.59%)	13973 (44.54%)	0.9216
Hemiplegia or paraplegia	1738 (11.08%)	3489 (11.12%)	0.8927

**Fig 2:**
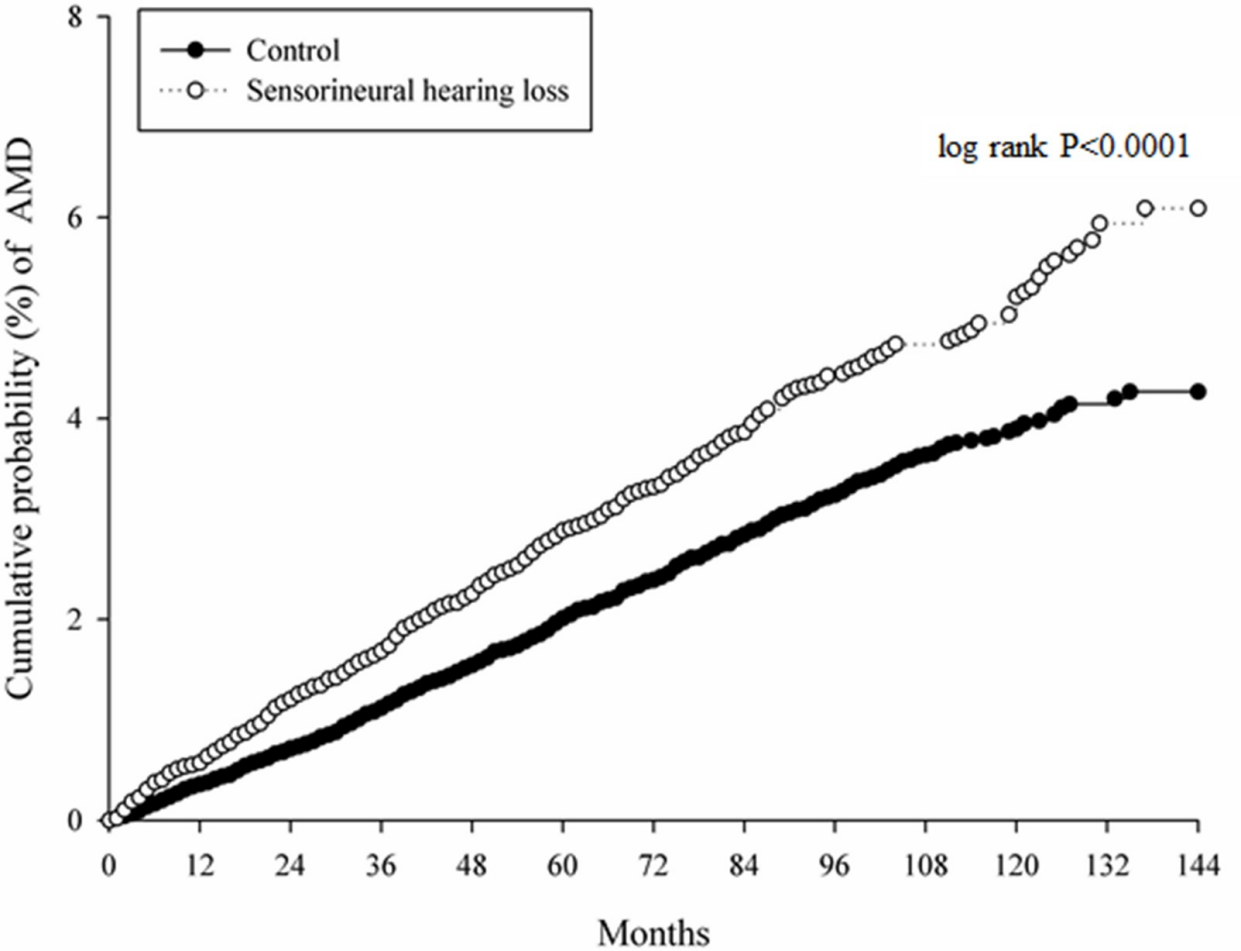
The Kaplan-Meier curve for the development of age-related macular degeneration. AMD: age-related macular degeneration.

After a study interval up to 16 years, there were 484 AMD events occurred in the study group and 660 AMD cases in those non-SNHL patients with a prominent crude relative risk (1.411, 95% CI: 1.255–1.587) in the study group (Table 2). Furthermore, the study group illustrated a significantly higher aHR compared to the control group after adjusting for multiple potential risk factors (aHR: 1.399, 95% CI: 1.244–1.574). Other prominent risk factors for AMD included aged 60–79 years old, aged more than 80 years old, ischemic heart disease, hyperlipidemia, Alzheimer's disease, liver disease and kidney disease (Table 3). Also, a higher cumulative probability of AMD was observed in the study group according to the Kaplan–Meier curve (log-rank *P* <0.0001, Fig 2).

**Table 2:** Incidence of age-related macular degeneration in the study and control groups.

	Study	Control
Median of follow up months	63	59
Follow up person months	1,022,293	1,964,658
New AMD case	484	660
Incidence rate* (95% CI)	47.34 (43.31–52.04)	33.59 (31.13–36.92)
Crude relative risk (95% CI)	1.411 (1.255–1.587)	Reference

* Incidence rate, per 100000 person months

AMD: age-related macular degeneration

CI: confidential interval

**Table 3:** Multiple Cox proportional hazard regression for estimation of adjusted hazard ratios on age-related macular degeneration.

Variable	aHR (95% CI)
SNHL (ref: Control)	1.399 (1.244–1.574)
Age (ref: 40–59)	
<40	0.191 (0.093–0.390)
60–79	3.954 (3.271–4.780)
> = 80	4.463 (3.537–5.630)
Sex (ref: Female)	
Male	1.104 (0.979–1.245)
Co-morbidities	
Hypertension	1.058 (0.915–1.224)
Diabetes mellitus	1.080 (0.948–1.230)
Ischemic heart diseases	1.233 (1.081–1.407)
Hyperlipidemia	1.200 (1.056–1.363)
Heart failure	1.083 (0.916–1.280)
Cerebrovascular disease	0.992 (0.865–1.137)
Dementia	0.905 (0.670–1.223)
Alzheimer's disease	2.257 (1.091–4.672)
Parkinson's disease	0.843 (0.537–1.325)
Liver disease	1.159 (1.025–1.310)
Rheumatic disease	1.041 (0.791–1.371)
Kidney disease	1.327 (1.173–1.502)
Hemiplegia or paraplegia	1.126 (0.957–1.326)

aHR: adjusted hazard ratio

CI: confidential interval

SNHL: sensorineural hearing loss

In the subgroup analysis stratified by age, gender and duration of SNHL, the risk of AMD was significantly elevated in all age subgroup and patients with different disease duration of SNHL. However, the incidence of AMD was similar in patients younger than 40 years old (Table 4).

**Table 4:** The sensitivity analysis for the adjusted hazard ratio of age-related macular degeneration stratified by follow up time, gender and age subgroups.

Subgroups	Incidence rate* (95% CI) of AMD		aHR (95% CI)
	Study	Control	
All	47.34 (43.31–52.04)	33.59 (31.13–36.92)	1.411 (1.255–1.587)
Follow up time (Year)			
<2	50.71 (43.63–58.94)	29.81 (25.93–34.26)	1.717 (1.399–2.108)
2–5	48.41 (41.85–56.00)	37.28 (33.08–42.01)	1.285 (1.064–1.552)
> = 5	43.49 (36.91–51.23)	34.25 (29.91–39.22)	1.246 (1.007–1.542)
*P* for interaction			0.7332
Gender subgroups			
Male	52.68 (46.94–59.12)	36.09 (32.63–39.91)	1.326 (1.137–1.546)
Female	41.17 (35.78–47.37)	30.74 (27.36–34.55)	1.255 (1.044–1.508)
*P* for interaction			0.6361
Age at index date			
<40	1.49 (0.37–5.94)	2.36 (1.06–5.26)	0.652 (0.131–3.239)
40–59	18.85 (14.88–23.86)	9.84 (7.79–12.44)	1.879 (1.347–2.622)
60–79	73.9 (66.12–82.60)	57.18 (52.24–62.59)	1.288 (1.116–1.487)
> = 80	100.98 (83.25–122.49)	64.19 (53.42–77.12)	1.585 (1.213–2.070)
*P* for interaction			0.1084

*Incidence rate, per 100000 person months

AMD: age-related macular degeneration

CI: confidential interval

aHR: adjusted hazard ratio

## Discussion

Shortly, the current study illustrated a significantly higher incidence of AMD in those diagnosed with SNHL compared to the non-SNHL individuals. Moreover, the aHR of developing AMD was still significantly higher in the SNHL population in the multivariable analysis. Other diseases that significantly related to the occurrence of AMD included Alzheimer's disease, certain cardiovascular diseases, liver disease and kidney disease.

There are several factors that correlated with the development of SNHL.[2, 13, 14] Age is a significant risk factor for the SNHL, in which the prevalence of SNHL was increased with older age in previous study.[9] In addition, both oxidative stress and inflammation process are also related to the development of SNHL.[15, 16] About the alternation of inner ear in the patients with SNHL, degeneration of hairy cell, the spiral ganglion cells and cochlear-nerve/hair-cell synapses were observed according to previous study.[17] In the aspect of correlated co-morbidities, several central nervous system lesions like Alzheimer’s disease, dementia and cognitive defect are associated with SNHL,[3] in which similar neurodegenerative features were found in both the SNHL and those brain disorders.[3, 7, 17, 18] On the other hand, age is also a prominent risk factor of AMD with higher prevalence in older population,[19] and oxidative stress and inflammation are also correlated to the AMD whether in dry or wet subtype.[20–22] Concerning the pathogenesis of AMD, damage of neuron and neuron-associated cells including retinal pigment epithelium degeneration, death of photoreceptor and thinning of retinal ganglion cell layer were found in AMD.[23–25] Moreover, AMD is also associated with central nervous system disorder such as Alzheimer’s disease and cognitive decline.[3, 26] Accordingly, we speculate that a vulnerable nervous system in both SNHL and AMD may exist since similar neurodegenerative features, risk factors and co-morbidities present in both diseases thus AMD will tend to develop in patients with SNHL, as illustrated by the results in the current study.

Concerning the relationship between SNHL and AMD, a previous study demonstrated a higher prevalence of SNHL with different severity in AMD population compared to non-AMD individuals.[12] In addition, dual sensory impairment including visual and hearing impairment is prevalent in elderly population while AMD and SNHL might account for the sensory impairment.[27] In the current study, the incidence of AMD in the individuals with SNHL is higher than those without SNHL with significant aHR and cumulative probability. To our knowledge, this is a preliminary experience to reveal the higher incidence of AMD in SNHL patients. Although the previous study already indicated a potential relationship between these two diseases, the study population in the previous study was too small with only 93 patients,[12] compared to the 47,058 individuals in the current study. Moreover, the longest follow-up period of the current study is nearly 11 years with the median follow-up interval of approximately 5 years, which is more adequate compared to the cross-sectional design in the preceding study.[12] Still, the SNHL-in-AMD trend in the previous study,[12] and the AMD-in-SNHL trend in the current study further strengthen the concept that a general weaken neurological system exists in those population.

About other significant risk factor of AMD revealed in the current study, the correlation between AMD and Alzheimer’s disease has been well-established according to preceding researches.[26, 28, 29] The cardiovascular diseases including ischemic heart disease and hyperlipidemia are also proven to be the risk factor of AMD,[30, 31] while the higher aHR of AMD in liver and kidney diseases might result from the worse health condition in the elderly which need further validation. In addition, all patients older than 60 years old showed a significantly higher incidence of AMD compared to patients aged 40–60 years and the patients younger than 40 years old revealed a significantly lower incidence of AMD compared to patients aged 40–60 years in the current study, which illustrates the significant effect of old age on the occurrence of AMD that corresponds to previous study.[10] The similar aHR among subgroup with different disease duration of SNHL indicated that AMD and SNHL results from vulnerable neurological system rather a causal relationship between them.

On the aspect of epidemiology, approximately 16 percent of population aged 20 to 69 years in US experienced SNHL. Further, the prevalence of AMD was estimated to be 6.8 percent in Caucasian population aged more than 40 years old and 13 percent in population older than 85 years old.[19] In the current study, the incidence of AMD was 47.34 per 100,000 person months which significant higher than the incidence of 33.59 per 100,000 person months in the control group. Moreover, the incidence of AMD in the study group is also numerically higher than the general incidence of AMD from the same population.[32] Since both SNHL and AMD affect a large percentage of population, a routinely ophthalmic examination can be recommended for patients that diagnosed with SNHL.

There are still several limitations in the current study. First, the retrospective nature of study design may reduce the homogeneity of patient population even after propensity score matching with multiple systemic diseases. Besides, we used claimed data rather than real medical documents, thus missing some important information like the severity and laterality of both the SNHL and AMD, and the result of the pure-tone audiogram as well as optical coherence tomography. In addition, we did not analyze the different type of SNHL (i.e. noise-related, age-related, medication-related, or idiopathic suddenly) separately. However, since all the SNHL shared similar neurodegenerative features,[1, 2, 33] this defect might influence the accuracy minimally.

In conclusion, the individuals with SNHL revealed a significantly higher incidence of developing AMD after adjusting multiple potential risk factors. Furthermore, the incidence of AMD was similar in patients with different disease period of SNHL. Further prospective study to evaluate whether different incidence of AMD exists among different SNHL severity is mandatory.
